# Flexible PCB Failures From Dynamic Activity and Their Impacts on Bioimpedance Measurements: A Wearable Case Study

**DOI:** 10.1109/OJCAS.2021.3122369

**Published:** 2021-11-24

**Authors:** SHELBY CRITCHER, TODD J. FREEBORN

**Affiliations:** Department of Electrical and Computer Engineering, University of Alabama, Tuscaloosa, AL 35487, USA

**Keywords:** Flexible printed circuits, wearable sensors, failure analysis, trace cracking, bioimpedance

## Abstract

Wearable health monitoring systems that collect data in free-living environments are becoming increasingly popular. Flexible printed circuits provide a commercially available option that can conform to the shape of a wearable system and support electronic sensing and flexible interconnect. However, repetitive dynamic activity can stress and damage the interconnect of flexible PCBs which degrades data quality. This case study evaluated the performance of flexible PCBs providing interconnect between electrodes and sensing electronics for tissue bioimpedance measurements in a wearable system. Resistance data (1 kHz to 128 kHz) was collected from localized knee tissues of 3 participants using the wearable design with flexible PCBs over 7 days of free-living. From electrical and optical inspection after use trace cracking of the flexible PCBs occurred, degrading tissue resistances reported by the wearable system. Exploration of these results advances understanding of how flexible PCBs perform in free-living conditions for wearable bioimpedance applications.

## I. INTRODUCTION

Wearable devices are becoming increasingly popular and serve to generate data from the human body in increasing volume, velocity, and variety. These devices provide the electronic sensing and circuitry to measure, store, and transmit data regarding movement, heart rate, respiration, blood glucose, blood oxygenation, muscle activity, and more through a variety of sensing modalities [1], [2]. Wearable devices are available in multiple form-factors including smart watches, rings, eyeglasses, forehead mounted sensors, and ear-worn monitors highlighting that these devices can be worn at different body sites for different applications [1]. The generation of personalized data from wearable devices provides the opportunity to support health related decision making using near-continuous physiological and health data collected during free-living [3], [4].

While an array of wearable sensing modalities are available, each have different on-body requirements. For example, inertial sensors can be placed on-body at a site of interest without direct tissue contact [5]. This can generate data regarding activity context, but does not provide details of what is occurring within the body at this location. Sensors that report physiological information (heart rate, blood oxygen, muscle activation) require direct contact with the tissue for their reliable data generation. This interface between skin and electronics is a critical site for the circuits and systems that underlie all wearable electronics. These systems require hardware and circuitry for: tissue-electronics interfacing, analog-front-ends for signal conditioning, conversions to digital values, data processing/storage/transmission, and peripheral circuitry for device interaction and supplying the necessary (and often low) power requirements. Focusing on the tissue-electronics interface, solutions must accommodate movement without degrading data quality. Available options for circuits and systems that accommodate movement include flexible printed circuit boards, conductive textiles, and stretchable conductors [6], [7], [8]. Of these options, flexible printed circuit boards (flex PCBs) are commercially available with design processes that utilize existing CAD tools, have a relatively low cost, and are available from a range of suppliers. Flex PCBs utilize printed conductive traces on a flexible substrate and can be integrated into a wearable design because of the ability to conform to the shape of the wearable and target on-body location. Common flexible PCB substrates include polyimide (PI) and polyester (PET) film with conductive material typically being copper (Cu), Cu foil, or a Cu alloy.

Flex PCBs have been investigated for a range of wearable applications, a small subset of which includes:

- Epidermal temperature monitoring in real-life conditions with a custom solution developed in-lab using an integrated circuit on a flexible PCB that contacted the skin surface through a stretchable patch [9];
- *In vivo* gastrointestinal mapping using a flexible printed circuit array of 32 electrodes interfaced to a rigid PCB [10];
- Gesture recognition using a flexible electrode array fixed to the forearm (with hydrogel electrodes) for electromyography measurements [11];
- Wrist and arm-wearable displays with flexible OLED panels, flexible circuit boards, and flexible batteries [12].

Further details on flexible circuits for healthcare monitoring applications for interested readers are available in the recent reviews by Chung *et al*. [13] and Salim and Lim [14].

Flexible PCBs are able to bend and twist increasing the range of environments in which they can be adopted. However, bending and twisting stresses these designs. Previous studies have analyzed the stress-strain relationship and failure-modes of flexible PCBs either through simulation or experimentation [15], [16], [17], [18]. However, there is still need to characterize the performance, time-to-failure, and failure modes of flexible designs during field use of wearable systems to inform if precise laboratory controlled tests are representative of real-world conditions and how data quality is impacted by free-living. This provided the motivation for this case study, to identify the effects of dynamic activity on the data quality of localized bioimpedance measurements collected using flexible PCBs integrated into a wearable system for knee tissue monitoring.

To assess the effects of dynamic activity on flexible PCBs, samples were electrically and optically inspected after either: i) 7 days of use in a wearable system by study participants during their free living or ii) twisting to emulate extreme movements in laboratory conditions. The following sections detail the testing processes for this case study, the sites of flexible PCB failures, and the impact on knee tissue resistance measurements collected/reported by the wearable system.

## II. WEARABLE BIOIMPEDANCE MEASUREMENTS

One sensing modality that requires direct tissue contact for measurement is electrical bioimpedance. This type of measurement characterizes the passive electrical behavior of a tissue which is related to the cell population, cell volumes, cellular membrane integrity, and the intra/extra cellular fluids. Bioimpedance is being investigated for health focused applications [19], [20]. Specific physiological features that bioimpedance is being used to characterize are: skeletal muscle fatigue [21], [22], [23], fluid shifts during dialysis [24], joint health [25], [26], blood pressure [27], and respiration [28].

**FIGURE 1.**
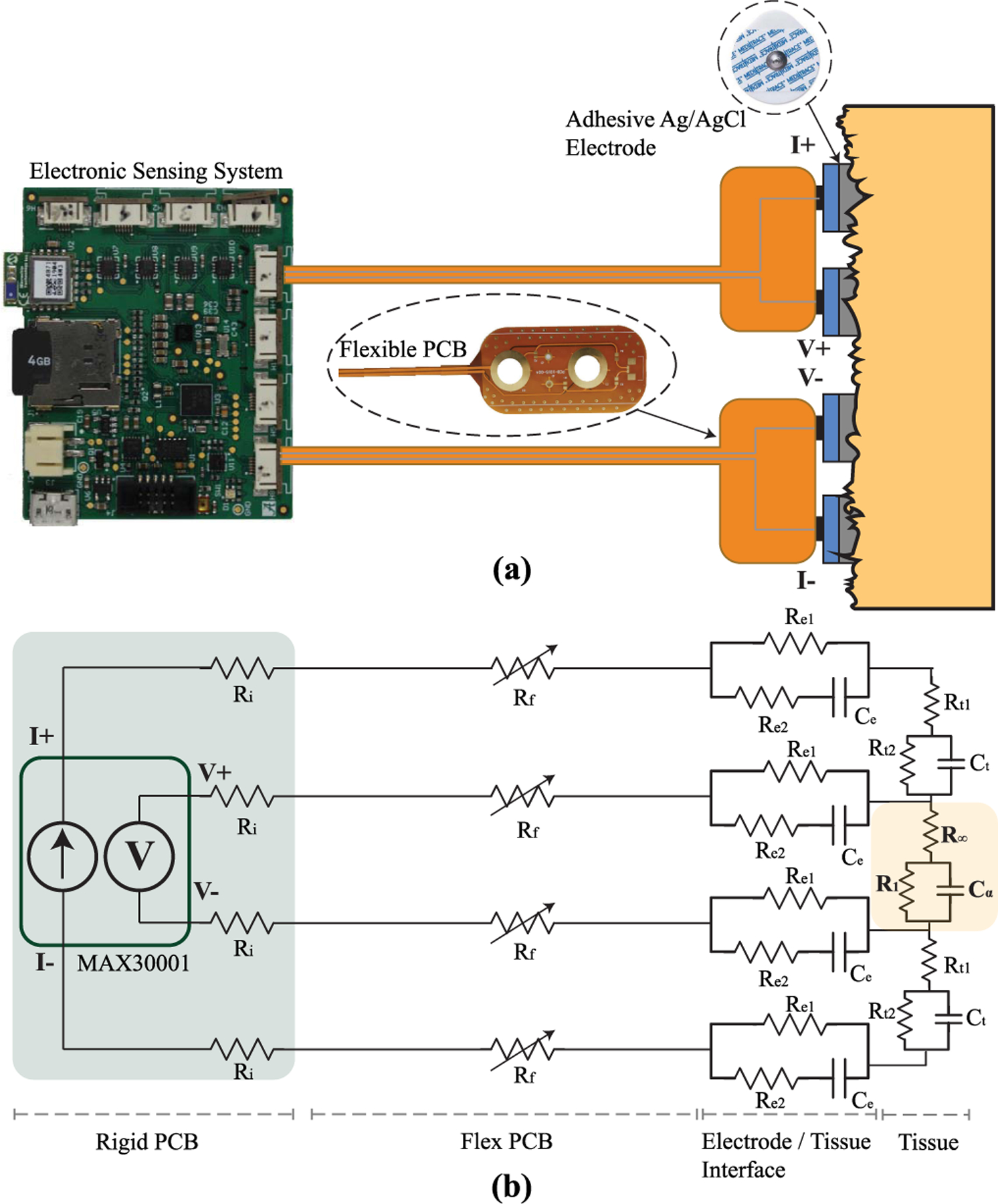
Tissue electrode interface for tetrapolar electrode configuration including a simplified electrical equivalent model.

Tissue bioimpedance is collected using bipolar or tetrapolar configurations, with two or four electrodes in direct contact with the skin. A tetrapolar configuration captures the transfer impedance between the excitation and sensing electrode pairs. A sample tetrapolar configuration of a wearable bioimpedance system interfaced to a tissue is shown in Fig. 1(a), highlighting the rigid PCB for the electronics, the flexible PCB for the interface, and the Ag/AgCl electrode connection to the tissue to be measured. With this configuration current is injected into the tissue using the excitation electrodes (I+, I−) and the voltage normal to the skin surface is measured using the sense electrodes (V+, V−). A circuit representation of the expected trace resistances in this tetrapolar system are detailed in Fig. 1(b). This representation includes the resistances from the rigid PCB (*R*_*i*_), interconnect resistance (*R*_*f*_), electrode/tissue interface (*R*_*e*1_, *R*_*e*2_, *C*_*e*_), residual tissue (*R*_*t*1_, *R*_*t*2_, *C*_*t*_), and measured tissue (*R*_∞_, *R*_1_, *C*_*α*_). Note that the interconnect resistance is represented as a variable resistor to denote that it is expected to change as a result of stress/fatigue during dynamic activity.

Tetrapolar configurations eliminate the tissue/electrode contact impedance (which is typically much larger than the tissue impedance) from the measured value. Though it is important to note that this impedance still impacts collection of the bioimpedance measurement. The tissue impedance measured by the voltage sensing electrodes is represented in Fig. 1(b) as a 2R-1C circuit (representing the observed frequency-dependent tissue behavior [21], [29]). The impedance expression for the 2R-1C model is given by:

(1) $$Z\left(j\omega \right)=R_{\infty }+\frac{R_{1}}{1+\left(j\omega \right)R_{1}C_{\alpha }}$$

The low and high frequency resistance is given by *R*_1_ + *R*_∞_ and *R*_∞_, respectively, with the transition between them effected by *C*_*α*_. Physiologically, these components are attributed to the intracellular fluid (*R*_1_ + *R*_∞_), total fluid (*R*_∞_), and the cellular membrane properties (*C*_*α*_) of the tissue. As a recent of example of efforts to utilize a similar model to represent physiological tissue changes, Fu and Freeborn reported alterations in equivalent circuit parameters representing biceps tissue bioimpedance after eccentric exercise that induced swelling and delayed onset muscle soreness in the days post-exercise [23].

**FIGURE 2.**
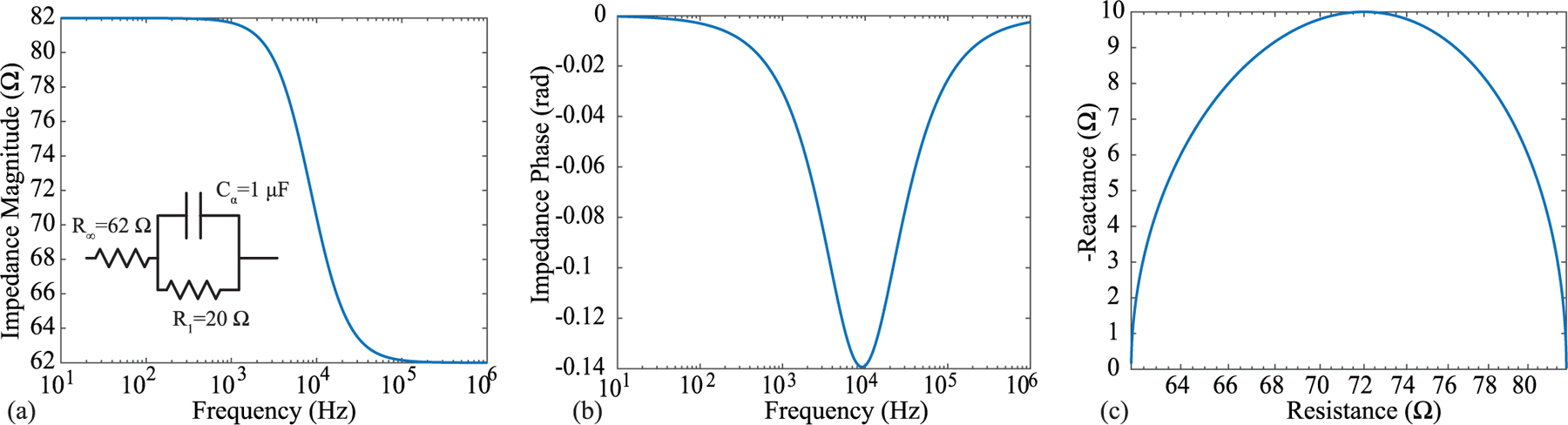
Ideal impedance magnitude of a 2R-1C model, often used to represent the frequency-dependent impedance of biological tissues, from 10 Hz to 1 MHz.

A sample of the impedance magnitude, phase, and resistance/reactance of (1) for fixed components values is given in Fig. 2. The specific component values for this ideal simulation are given in the circuit subset. From Fig. 2(a), the impedance magnitude at less than 1 kHz is 82 Ω (*R*_1_ + *R*_∞_) and the impedance magnitude at 100 kHz is 62 Ω (*R*_∞_). Referring back to Fig. 1, additional 2R-1C models are used to represent the electrode/tissue interface (*R*_*e*1_, *R*_*e*2_, *C*_*e*_) and the tissue impedances not captured by the voltage sensing electrodes (*R*_*t*1_, *R*_*t*2_, *C*_*t*_). These highlight that there are impedances present in the configuration setup that can impact the measurement but are not captured in the measurement.

Circuits and systems designers are often focused on the sensing circuitry of bioimpedance measurements. Research efforts often target improving the circuitry for current injection, voltage sensing/amplification, and filtering [30], [31]. However, it is also important for designers to understand the interface of the circuits and systems to the biological tissue and how it can impact their measurements. The interface of electronics to tissues for bioimpedance applications is often achieved using silver/silver-chloride (Ag/AgCl), textile, or metal electrodes [32], [33], [34]. Ag/AgCl electrodes are available with and without adhesive gels referred to as “wet” electrodes and “dry” electrodes, respectively. The addition of gel improves contact and charge transfer between electrode and tissue and are widely utilized for bioimpedance studies [21], [26], [28], [35]. “Wet” electrodes have lower interface impedance [32] and improved contact with the tissue as a result of the gel filling surface irregularities compared to their “dry” counterparts. However, “wet” electrodes cannot be worn for extended periods of time due to oxidation and drying, limiting them to days of use before requiring replacement.

Dry electrodes can enable data collection continuously without regular replacement due to gel/adhesive drying, which will enable wearable designs with greater usability and unobtrusiveness. Due to this potential, efforts are ongoing to evaluate dry electrode designs fabricated using commercial rigid [36], [37] and flex [38], [39] PCB processes and their performance for biosensing applications. Though using dry electrodes has additional challenges (compared to their wet counterparts) such as reduced contact quality, larger electrode/tissue impedance, and increased susceptibility to noise and capacitive leakage. Most research explores the electrode characterization in static conditions, but when integrated into wearable designs electrodes will be in dynamic conditions subjected to mechanical stresses, fatigue, and damage. While previous studies have characterized the failure modes and alterations of flex PCB copper traces, there is little experimental data regarding how alterations in copper features of a flex PCB effect data quality of sensing systems in dynamic, real-world conditions. This motivates the case study that is presented here.

### A. FLEXIBLE PCB FOR TISSUE/ELECTRODE INTERFACE

A flex PCB for tissue/electrode interfacing must provide contact to the tissue and interconnect to the sensing electronics. This case study focuses on quantifying the effects of interconnect alterations of a flex PCB during dynamic activity and remove dry electrode effects as a confounding variable. To achieve this, a flexible PCB that interfaced Ag/AgCl electrodes to the corresponding excitation and measurement signals of a bioimpedance sensing system was designed/fabricated. Fabrication utilized a commercial PCB manufacturer to evaluate the performance of a low-cost design that could be quickly fabricated without additional customization steps such as additional gold plating [38] or chlorination [36]. While additional customization steps increase performance and functionality, they require additional materials expertise and equipment which may not be available to circuit designers. The choice to use only features available commercially in this work was done to represent a typical use-case of a circuits and systems designer.

**FIGURE 3.**
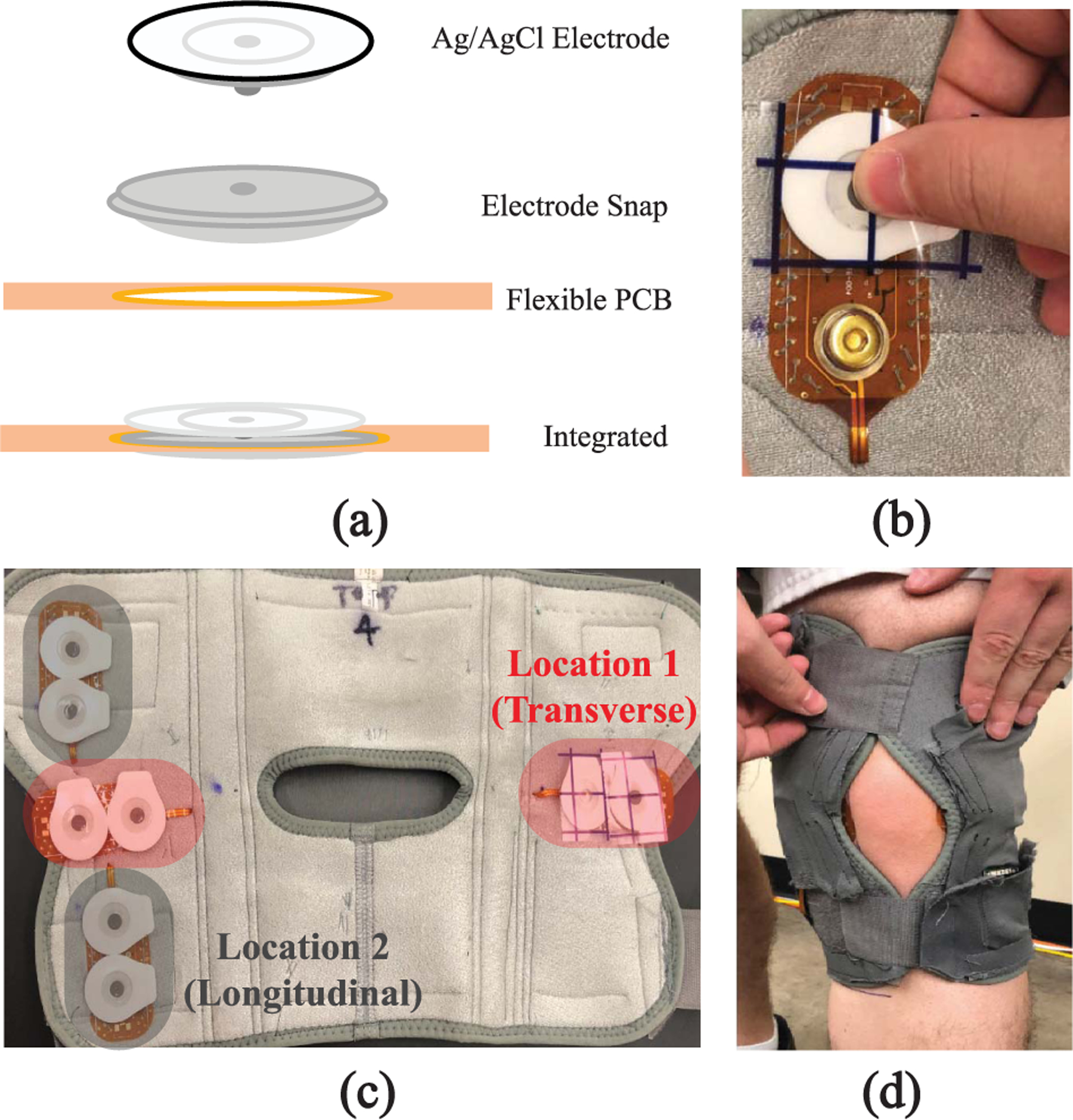
Overview of flex PCB interfacing with both electrodes and wearable system. Specifically, (a) stack-up of flex PCB / electrode connection, (b) sample of Ag/AgCl electrode snapped into design, c) integration 4 flexible PCBs into wearable design to support 2 tetrapolar configurations, and (d) placement of the wearable on the knee of a study participant.

The flex PCB in this work, shown in the subset of Fig. 1(b), paired a current injection and voltage sensing electrode to facilitate tetrapolar configurations when two are utilized. Note, there are additional component pads (e.g., resistor, cable connector, testpoints) on the flex PCB which provided test points for bench-top testing, but were not used in this study. The flex PCB was fabricated using a standard 2 layer flexible printed circuit stack-up (0.23 mm) with an electroless nickel immersion gold (ENIG) surface finish. ENIG was chosen because copper is not bio-compatible [40] and because ENIG is widely available at low cost from manufacturers. Other gold finishes (i.e., soft and hard gold plating) are also available which could be investigated in future designs. Figure 3(a) visualizes how the Ag/AgCl electrode is connected to the flexible PCB. An electrode snap was soldered to a 3 mm ring to provide the mechanical feature for an Ag/AgCl electrode to be snapped into the design for the electrical connection. An Ag/AgCl electrode snapped into the design is shown in Fig. 3(b)). The traces routed to the electrode snaps were 0.4 mm wide. The flexible PCB subset in Fig. 1(a) shows the “tail” to interface to the rigid PCB sensor system. This section was 50 cm long with 1 mm traces. The traces had exposed copper (with an ENIG surface finish) every 6 − 8 cm for customizable cable lengths (achieved by cutting at the desired length). All traces used 1.0 oz copper. Stiffener (0.1 mm) was placed opposite the exposed pads to increase cable thickness for a tighter connection when inserted into the flat flexible connector (FFC) on a rigid PCB which contained the system electronics. Note, that the perimeter vias in the design were included to stitch and fasten the PCB to a textile.

## III. FREE-LIVING DATA COLLECTION

To assess bioimpedance data quality collected using the flex PCBs throughout periods of dynamic activity, localized knee tissue bioimpedance was collected from 3 participants (2 males, 1 female, average age 26 years) over 7 days while wearing the wearable knee sensing system. Prior to their participation, each study participant provided their written informed consent to be in the study. The participants reported no history of knee pain and no previous knee injuries. This research and its activities were approved by The University of Alabama’s Institutional Review Board (UA IRB-18-013-ME).

### A. WEARABLE KNEE SENSING SYSTEM

The wearable system utilized 4 flexible PCBs sewn into a commercial knee brace to collect longitudinal and transverse bioimpedance measurements. The knee was investigated because it is a joint with significant movement during activities expected during daily living (sitting, walking, stair climbing). A knee brace was selected as the mechanical platform (as opposed to affixing the electrodes to the tissue with medical adhesives) because textile braces are already widely worn adopted by persons with knee injuries or need for knee support, which is likely to improve adoption of knee monitoring technologies if integrated into this form factor.

The locations of the flex PCBs in the knee brace (with connected Ag/AgCl electrodes) are shown in Fig. 3(c). These two sites were selected because they capture tissue resistance of the knee joint (Location 1) and knee joint/skeletal musculature for joint motion (Location 2), with higher movement expected for Location 1 due to the significant knee joint activity in all lower body movements. The flexible cables were routed on the brace exterior to the sensing system (a rigid PCB) which was secured in a pocket attached to the brace. The sensing system utilized a Texas Instruments MSP432 microcontroller to control a MAX30001 IC, a single-chip integrated circuit (IC) with analog-front-end for bioimpedance measurements, and additional analog multiplexers to measure the impedance from both knee sites [41]. The MAX30001 IC has the capability to collect both the real and imaginary components of impedance (resistance and reactance respectively), but not both simultaneously. The MAX30001 was configured to use an excitation current of 8 *μ*A and resistances were collected at 8 discrete frequencies from 1 kHz to 128 kHz at an approximate rate of 1 measurement every 3 minutes. All collected data was stored on an on-board microSD card. Additional details regarding the sensing system and its validation using known impedance test loads are provided in [41], [42], [43].

Prior to data collection, each participant was trained on the installation of the knee brace. Specific instructions included to replace the Ag/AgCl electrodes every morning, wear the brace for > 10 hours per day, and remove the brace after each full day. A sample of the knee brace installed on a participants knee is given in Fig. 3(d). After the 7-day trial, the data was downloaded by the study team for further processing.

### B. PARTICIPANT KNEE TISSUE RESISTANCE

**FIGURE 4.**
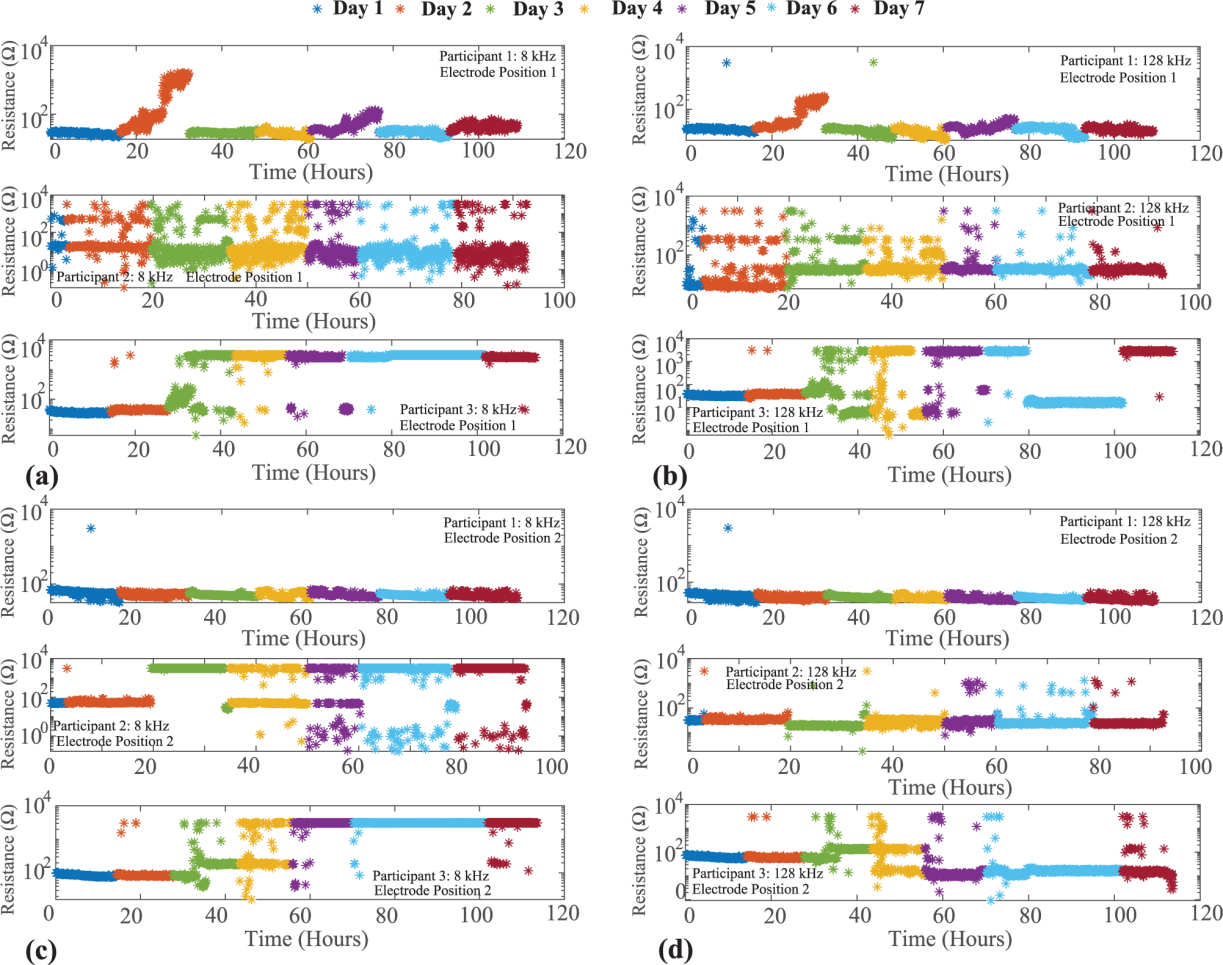
8 kHz and 128 kHz resistance of two knee sites collected using flex PCBs integrated into wearable system from 3 participants across 7 days. Measurements from Participant 1 represent a reliable set of resistance data using the flex designs with Participant 2 and 3 representing unreliable data expected to be a result of fatigue/cracking of the flex PCBs.

The knee tissue resistance collected for all 3 participants across 7 days is shown in Fig. 4. Figures 4(a) and (c) detail the 8 kHz resistance for the two knee locations and Figs. 4(b) and (d) detail the 128 kHz resistance. The colored markers represent the day that data was collected (with a unique color for each day). Participants wore the brace from 90 hours to almost 120 hours across the 7 days with more than 1800 measurements at each frequency collected. To visualize the resistance range a logarithmic scale is utilized in Fig. 4. With the exception of 1 datapoint the resistances from Participant 1 (electrode position 2 in Fig. 4(c)) are < 100 Ω. This range aligns with previous reports of localized tissue resistance from the literature, summarized below:

- Approximately 70 Ω to 100 Ω for 50 kHz resistances of lower and upper limb muscle groups of young and older adults reported by [44];
- Approximately 37 Ω to 68 Ω for 50 kHz resistances of lower limbs of injured and non-injured football players by [45];
- Approximately 27 Ω to 46 Ω in the range from 10 kHz to 100 kHz in studies of localized bicep tissues before and after fatiguing activity by [21];
- 45.87 ± 12.77 Ω and 46.26 ± 13.71 Ω for 50 kHz resistances of the right and left (transversal) thigh during test-retest studies by [46].

Additionally, the 128 kHz resistances have lower values than the 8 kHz resistances, which is expected based on the frequency dependent behavior of tissues which can be modeled using (1).

**FIGURE 5.**
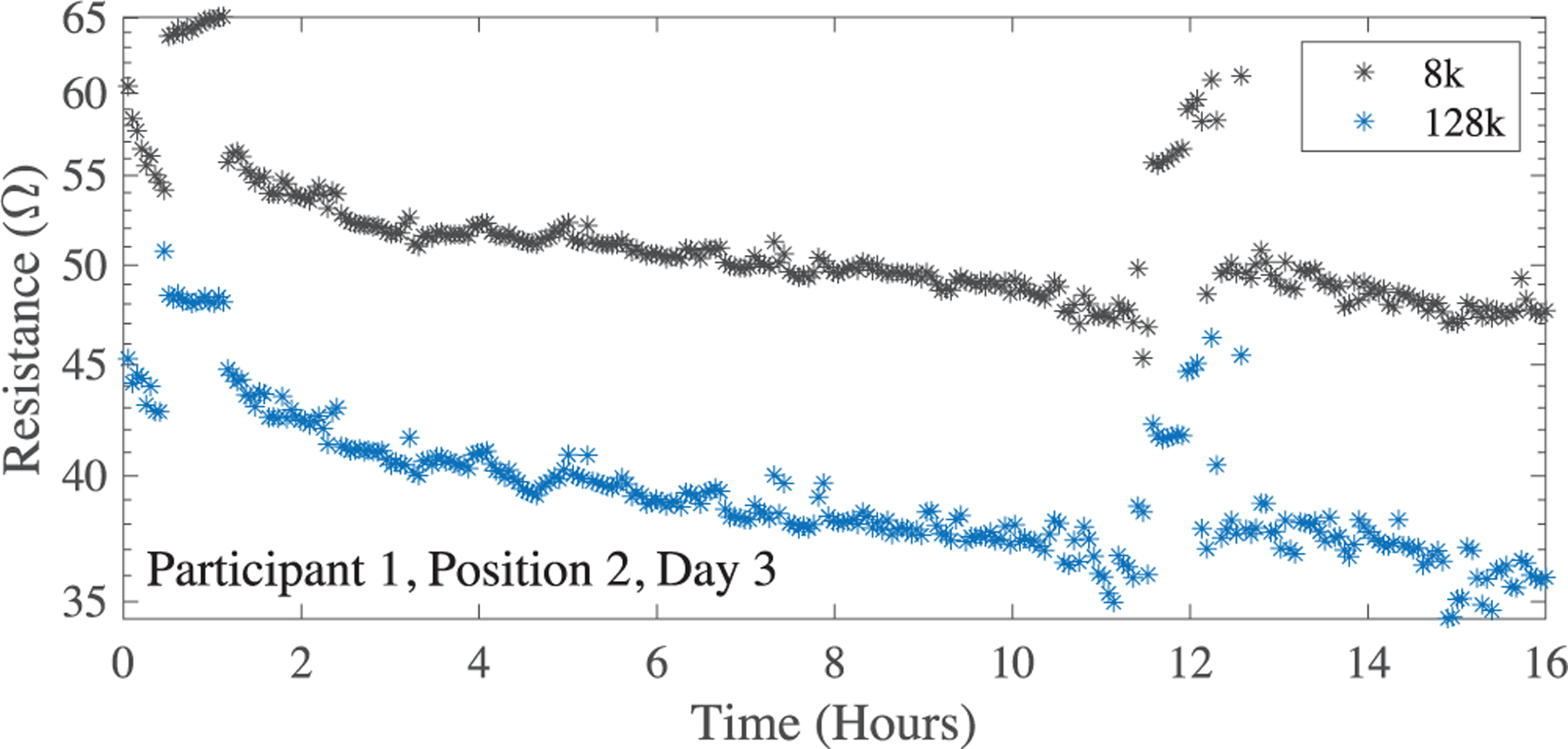
Sample of a single day of high quality resistance data, collected from Participant 1 on day 3 using the position 2 electrodes.

These features (appropriate resistance magnitude/range and frequency behavior) support that the flex PCBs provided reliable interconnect during measurements for this particular participant/knee location across the 7-day period. With the exception of day 2 data (which as an increase in resistance above 1 kΩ) this was also observed for electrode position 1 for this participant. To highlight the resistance variation across a day with reliable data collection (which cannot be observed using the logarithmic scaling in Fig. 4), day 3 data from participant 1 (electrode position 2) is shown in Fig. 5. Across this day, both 8 kHz and 128 kHz resistances are within an approximate 15 Ω range.

While data from Participant 1 supports that the flex PCBs were reliable for data collection in the wearable system, indicators of reliable data collection were not observed for Participants 2 and 3 across all days. Reviewing Participant 3 data, resistances across days 1 and 2 are < 100 Ω (with only a few exceptions). But from day 3 to day 7 the range and inter-day variability in resistances (for both knee sites) increases significantly. Reviewing the data from Participant 2 (Electrode Position 1), there are consistent variations in measurements from < 10 Ω to > 1 kΩ across all days. Similar trends are observed in data from days 3 to 7 of electrode position 2 data. The is a range that has not been observed/reported in other studies of tissue bioimpedance collected using tetrapolar configurations. As a result, these measurements are expected to be a result of the measurement system and not representative of the tissue.

To eliminate the sensing electronics as the source of error, the sensing system operation/accuracy was confirmed by measurements of an on-board 2R-1C model realized using discrete components of known value integrated on the rigid PCB design. This model is independent of the tissue/electrode interfacing circuitry of the flex PCB. Measurements within the expected range of the discrete component values eliminated the operation of the MAX30001 or other electronics in the sensor system as the cause of the unreliable resistance data. This supports that flex PCB traces are fatiguing and cracking, leading to degraded data due to unreliable connections for the excitation and sensing signals. It is important to consider here that the flex PCBs in the brace for Participant 2 were the same as Participant 1, meaning they had accumulated more than 110 hours of use/wear before the second set of data collection with Participant 2; which may account for the degraded data that occurred almost immediately with this participant.

### C. ELECTRICAL AND OPTICAL INSPECTION OF FLEX PCBS

**FIGURE 6.**
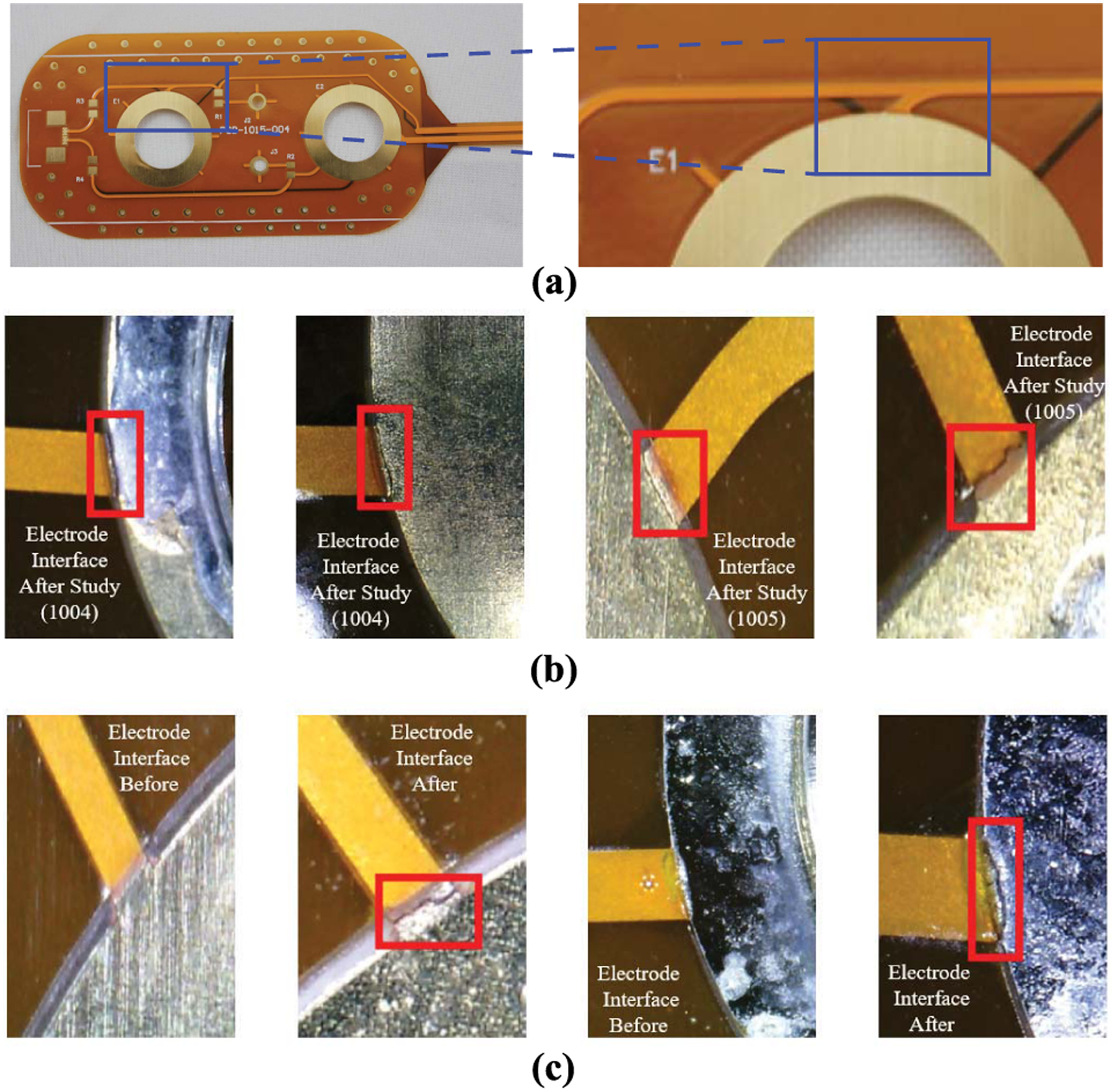
(a) An example location of the interface where the trace cracking occurred, Optical images of flexible PCB cracks at the electrode snap interface (b) after data collection in wearable knee brace and (c) fatiguing through multiple twisting cycles. Red boxes highlight the crack locations.

Due to the change in data quality (e.g., increase in variability and data above the expected values for biological tissues) it was hypothesized that micro-cracking of traces on the flexible PCB were the primary cause. Referring back to Fig. 1(a), fatigue and damage to the interconnect resulting in increasing *R*_*f*_ such that there was limited to no electrical connectivity. To test this hypothesis, the flex PCBs were electrically and optically inspected after removal from the braces. For electrical testing, continuity between the snap electrode and the trace inserted into the rigid PCB header of the sensing system was assessed using a Keysight U1232A digital multi-meter. For optical inspection, an Aven tools digital microscope captured magnified images of all signal traces on the flex PCB for signs of cracking or other damage (such as coverlay damage or fatigue). An example location of the cracking on the flexible PCB is emphasized in Fig. 6(a) with magnified images of cracked traces shown in Fig. 6(b). The red boxes in these images highlight the cracked area. From the optical inspections, all of the trace failures occurred at trace-to-pad interfaces and not at any locations with polyimide; nor was there observed damage to the polyimide coverlay. The flexible PCBs from Participants 2 and 3 all failed continuity testing, supporting that the dynamic activity of the wearable knee system resulted in damage to the flexible PCBs. This damage is expected to be the source of the high variability of the reported data (and will be explored further in the discussion). Note, the same flexible PCBs and cables were used for Participants 1 and 2. Cables were reused because the set of 4 flex PCBs/cabling passed electrical continuity testing after use by Participant 1. This supported the continued use of these electrodes during the data collection with Participant 2. Participant 3 utilized a new set of 4 flex PCBs for their data collection, replaced because of the electrical continuity failure during testing after use by Participant 2.

## IV. REPLICATION OF FLEXIBLE PCB FAILURE

To validate that copper trace cracking was the primary source of errors in the free-living data collection, additional flexible PCBs were used to collect measurements of a known impedance load (the 2R-1C topology given in Fig. 2) before and after exposure to stress using repetitive extreme movements. Additionally, both electrical and optical inspections were conducted on the flexible PCBs throughout this stressing period. Electrical continuity measurements were collected in the following sequence: baseline (prior to fatigue/stress), measurement after one fatigue/stress cycle, measurement after every additional 5 fatigue/stress cycles until failure or until 96 cycles were reached. Continuity measurements were recorded as either a pass or fail. Optical inspection of the PCBs were performed prior to any fatigue cycles and after the fatigue testing had been completed.

**FIGURE 7.**
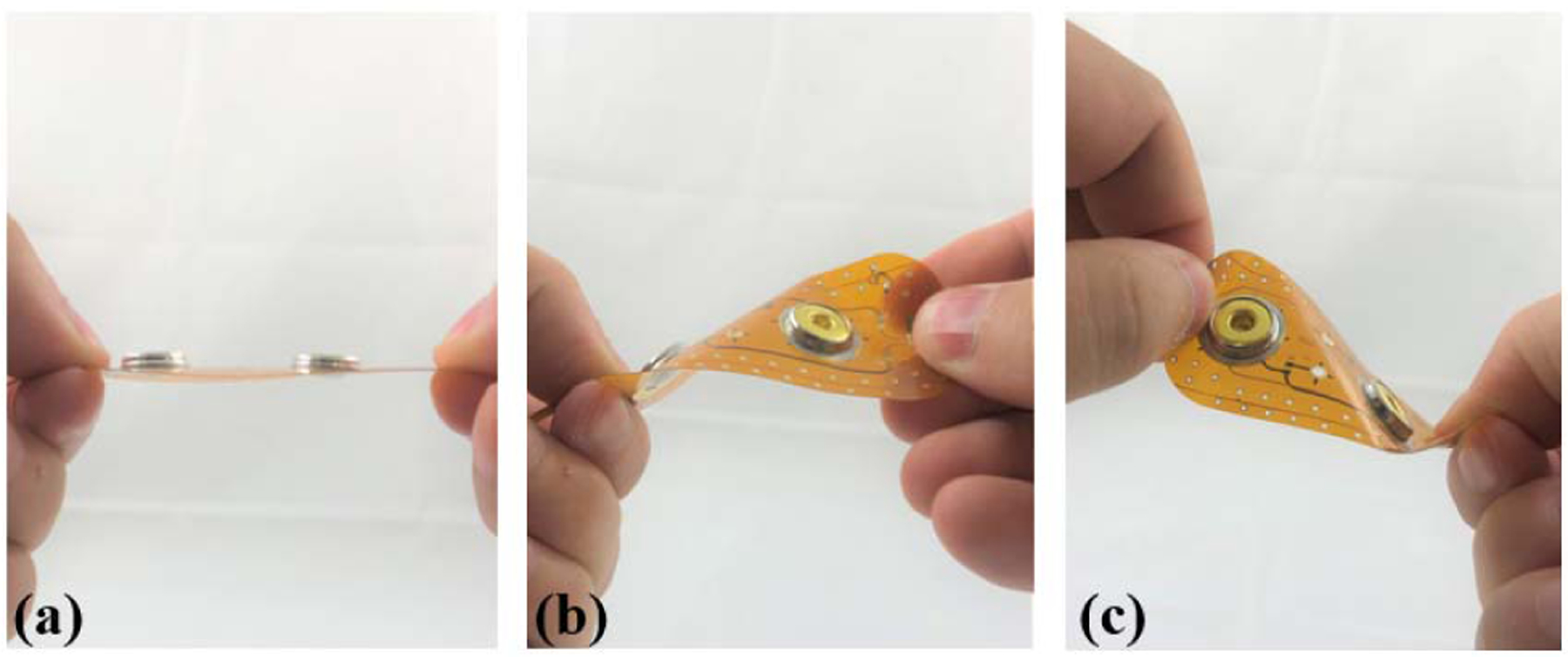
Progress of twisting movement applied to fatigue and crack flexible PCB beginning. A single twisting cycle started at position (a) transitioning through (b) and (c).

The extreme fatigue utilized twisting of the flexible PCB, shown in Fig. 7, to stress the design. For this twisting process the PCB was held flat horizontally (Fig. 7(a)), then twisted forward (Fig. 7(b)) and backward (Fig. 7(c)). This motion was utilized to emulate extreme cases of motion that the flex PCBs at location 1 could experience (in comparison to the single-axis bending motion typically utilized to stress designs). The aim of this testing was not to determine the specific number of twisting cycles that would result in a crack, it was only to cause a trace crack on the flexible design. The continuity testing and visual inspection was focused on the electrode interface location because this is where cracking from the wearable data collection was observed.

This testing cycle was completed using 4 flex PCBs, two each with and without the electrode snaps. This was done to determine if the addition of the electrode snap and solder to the annular ring on the flexible PCB affected the inspected failure profile. Both sets of flexible PCBs failed the electrical continuity tests after the fatigue cycling of less than 100 cycles. A sample of one of the flexible PCBs before and after the twisting is given in Fig. 6(c). In this figure, the red box outlines the observed cracks (which were not present prior to testing). Also, these cracks are visually similar and match the locations (e.g., trace-to-pad interface) compared to the inspection of the flex PCBs in Fig. 6(b) which detail the failures during free-living. These results support that the cracking of traces of the flexible PCBs can result from twisting (expected to occur in the knee brace) and that this cracking does lead to continuity failures of these traces. Further, the failure of the design with and without the snap connector supports that the failure was not a result of only the mechanical snap in the design.

### A. TRACE CONTINUITY DURING MOVEMENT

**FIGURE 8.**
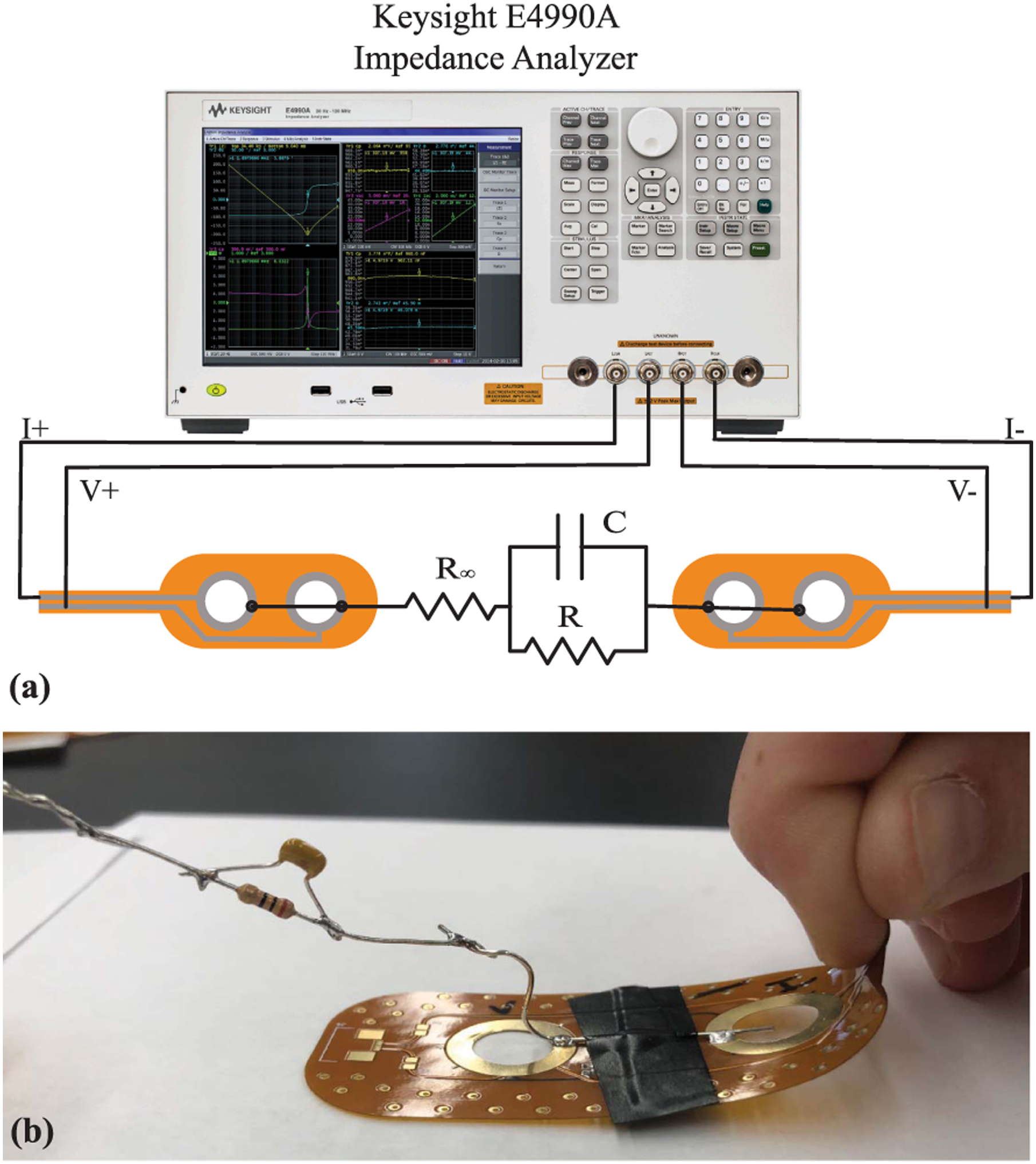
(a) Experimental setup to measure impedance of a 2R-1C model using the flexible PCBs and (b) the bending motion applied to the flexible PCB to test if cracked traces could make electrical connections for measurements.

**FIGURE 9.**
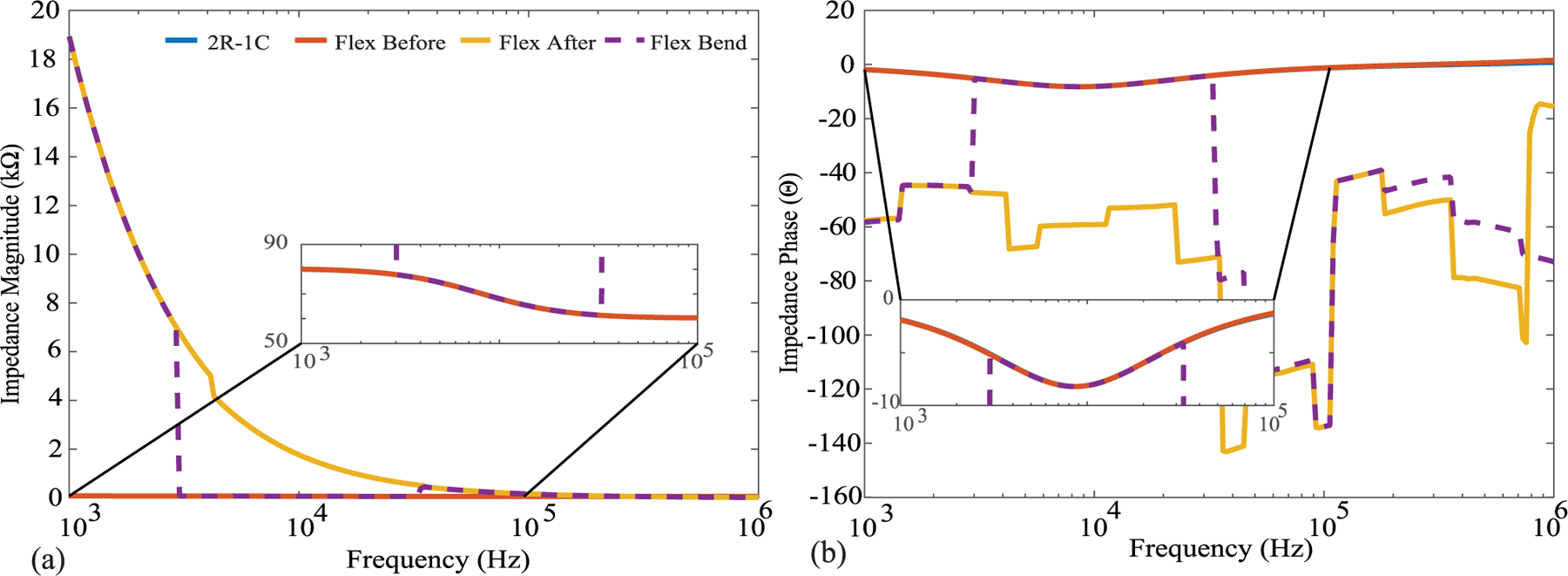
(a) Impedance magnitude and (b) phase measurements of 2R-1C test load collected using Keysight E4990A impedance analyzer interfaced to test load: directly (blue), using flexible PCBs before fatigue cycle (red), using flexible PCBs after fatigue cycle (orange), and using cracked trace flexible PCBs with applied pressure (dashed).

During electrical continuity measurements the placement of the multi-meter probe impacted the test result (e.g., pass or fail). That is, applying pressure with the probe near the location of the cracked trace resulted in a successful measurement of continuity while pressure far from the cracked trace resulted in failed continuity. This was attributed to pressure resulting in temporary contact of the cracked trace yielding electrical continuity. To validate, two flexible PCBs were modified to interface to a 2R-1C model (*R*_∞_ = 62 Ω, *R* = 20 Ω, *C* = 1 *μ*F) that would emulate a localized tissue impedance for measurement by a Keysight E4990A impedance analyzer. A sample of this test configuration is detailed in Fig. 8(a). In this configuration measurements of the test impedance (magnitude and phase) were collected prior to any fatigue cycling, after fatigue cycling (which induced a cracked trace), and when applying pressure to the cracked trace. These three sets of measurements (from 1 kHz to 1 MHz) in comparison to direct measurement of the 2R-1C model are given in Fig. 9. The direct measurement and measurement with the flexible PCBs (prior to trace cracking) show perfect magnitude and phase agreement. Observing the measurement after the fatigue cycling the impedance from 1 kHz to 10 kHz has magnitude greater than 1 kΩ (that decreases with increasing frequency) with a negative phase angle. This impedance reflects the parasitics in the test configuration and leakage paths between cabling and fixtures and dominates the bipolar measurements after the trace cracking and loss of a low resistance connection to the test load. For the final measurement pressure was applied to the trace by flexing the PCB (shown in Fig. 9(b)). This motion was applied for a short duration which based on the stepped-sine measurement method of the Keysight E4990A resulted in a subset of the frequency sweep having electrical continuity. This specific measurement is presented in Fig. 9(a) as a dashed line. Notice that the measurements from 3 kHz to 30 kHz show strong visual agreement with the direct measurements of the test load. Therefore, even with a cracked trace that degrades measurements during static conditions, dynamic activity and changes in applied pressure can yield accurate measurements. This may present an opportunity to develop algorithms that can both identify when trace cracking has occurred and what data during this period may still be considered reliable for further processing and interpretation.

## V. DISCUSSION

The flexible PCBs integrated into a wearable knee brace yielded reliable knee tissue resistance from only one of three study participants in this preliminary trial. The degraded resistance data was attributed to cracking traces occurring at trace/pad transitions on the design. Considering the circuit representation of the system in Fig. 1 this cracking represents a significant increase of *R*_*f*_ from less than 0.5 Ω (estimated for a 0.4 mm trace) to an open circuit which interrupts the application of the excitation current or appropriate voltage sensing in this bioimpedance system. Both of which prevent the measurement of the tissues and is reflected in the high value resistances (> 1 kΩ) and high variability. The high variability is attributed to the sensitivity of the voltage sensing circuitry to external influence and the high-values to the saturation of the signal conditioning circuitry of the MAX30001.

While trace cracking of a flexible PCB during flexing and twisting is not unexpected, reports have detailed failures of copper traces after thousands of machined bending cycles [17], [47], [48], the interest here is in how these failures impact reported data (which can support development of improved processing algorithms for failure identification and data cleaning) and recommendations to improve flexible designs for use in movement intensive applications.

No evidence of trace damage was observed on copper traces covered by polyimide on the flex PCBs analyzed in this study. Similar results were reported during another investigation of fatigue behavior of bare and polyimide covered Cu foil [17]. In that effort, the number of cycles to failure increased from hundreds to thousands with the inclusion of polyimide [17]. Additionally, another study of trace reliability reported mean cycles to trace failures (defined as increases of 20% or greater for trace resistances) of 585 and 1022 cycles for tension and compression based cycling, respectively [47]. Noting that the location of the failure was at trace-to-pad transition regions, which was also noted in the failure analysis of this work and the optical images of Fig. 6. Therefore, future designs should include polyimide over traces and trace/pad transitions such as the annular rings on the flex design in Fig. 3(a). Increasing the coverage of polyimide at this location is hypothesized to reduce the stress on the copper at this transition (which was the only site of failure on this design) and increase potential time to failure from activity (but requires further study to confirm). Future studies should investigate using teardrops at interconnection points between exposed copper and polyimide covered Cu foil for potential improvements in flexible PCB life.

An interesting observation is the difference in data quality between participants of the study. Participant 1 had approximately 110 hours of use/activity in the wearable design with no data quality changes attributed to cracked traces while Participant 3 appears to have had trace failures after 30 hours of use/activity. These differences may be a result of different activity profiles and contact differences of the electrode/flex PCB assembly with the participant knee (which could apply stress to different areas of the design and traces). Considering that the twisting motion applied to flex PCBs in this study resulted in trace failures after < 100 cycles it is likely that this type of movement in free-living resulted in faster times-to-failure compared to the bending only motions studied by Lall *et al*. [47]. However, this requires further study to evaluate with the addition of movement data to quantify associations of time-to-failure with the amount, intensity, and patterns of movement.

The impact of dynamic activity after copper trace cracking introduces another challenge for bioimpedance systems using flexible PCBs. This challenge is identifying the cause of failure (e.g., component failure, interconnect failure, interface failure) since different failure modes require different solutions (some which can be resolved by the user). While movement profiles do stress the traces on a flexible design, the movement of the wearable can also result in disconnection of the electrodes from the tissue surface. An electrode disconnect event also prevents accurate measurement of tissue impedance and can yield high-variability data similar to that observed for Participant 2 and 3 data in Fig. 4. Consider the data for Participant 2 in electrode position 2 which has low variability in days 1 and 2, supporting that reliable data collection occurred for this period. Inspecting the day 3 resistance, the 8 kHz data has differences of more than 1000 between data collected minutes apart. This pattern of resistance data oscillating between values that are within and then exceeding the range expected for biological tissues could indicate a cracked trace that is periodically connected during movement. This is supported by the measurements presented in Fig. 9, which showed how movement of the PCB can result in accurate measurements even with a damaged trace. This collection of unreliable resistance as a result of flex PCB failures can have a significant effect on processing and interpretation of bioimpedance data. Consider the ratio *h*_*α*_ proposed by Mabrouk *et al*. [30] as a potential metric for differentiating healthy and injured ankles:

(2) $$h_{\alpha }=\frac{\Delta R_{100\text{ kHz}}}{\Delta R_{5\text{ kHz}}}$$

where *R*_100 kHz_ and Δ*R*_5 kHz_ are the range of resistances at 100 kHz and 5 kHz, respectively, measured across a fixed window of time. Corrupted resistance values in either frequency could cause a significant skewing of this metric and its interpretation. Further, consider the equivalent circuit fitting utilized by [23] as a potential metric for identifying fatigue-related changes in skeletal muscle. Using this approach, optimization solvers determine the set of circuit parameters that best fit a selected model to the experimental data. Corrupted data will impact the outcome of optimization solvers for this type of analysis and need to be identified and removed prior to using the solver.

On review of the results in Fig. 5, it is important to note the variability of tissue bioimpedance that occurs throughout daily living. Note that in Fig. 5 there are the significant increases in tissue resistance at approximately 1 hour and 12 hours. While this data cannot elucidate the mechanism of change, it may result from changes in body position and accompanying fluid shifts in the knee. Reports of fluid shifts due to postural changes in segmental body regions have been reported by Fenech and Jaffrin, occurring quickly in the minutes after the postural change [49]. This is important when interpreting data from free living and supports that processing algorithms need to consider both current body position (which has been done by in previously reported systems for knee monitoring [25]) and the previous body positions since position alterations may cause alterations greater than the changes associated with a joint injury or long time scale alteration. This serves to highlight why wearable systems, such as the one presented in this work, are needed to quantify tissue resistances across both short (minutes/hours/days) and long (weeks/months) timescales.

Returning to the sources of errors, it should be acknowledged that the resistance variability observed in this effort could be attributed to trace failure, electrode disconnection, or a combination of both. This highlights a limitation of using only the physiological data for decisions regarding system state of performance. This supports the need for self-monitoring by wearable devices and informs a path forward to improving system reliability for circuits and systems designers. While recent bioimpedance integrated circuits such as the MAX3000x include on-chip testing for electrode disconnect events [41], there is an opportunity to both expand self-test methods to identify trace failures for real-time reporting and resolution and develop automated methods of correction/compensation/self-healing. This will require new hardware designs that generate multi-modal data (e.g., bioimpedance, activity profiles, trace health, electrode status), multi-modal data fusion, processing for real-time assessment/reporting, and approaches to redundant hardware. The trace alterations in this effort also highlight another test site for the underlying sensing circuitry in wearable sensing systems. Characterization of the accuracy and performance of circuitry is completed in static conditions. That is, in reference to Fig. 1(b), values of interconnect resistance (*R*_*f*_) are fixed and low-value (< 1 Ω) while the electrode/tissue interface is not included at all (e.g., *R*_*e*1_ = 0 Ω). This represents an ideal situation which is useful to characterize a device, but designers should consider additional testing to evaluate the range of *R*_*f*_ and electrode/tissue interface values that their circuits/systems can operate without degrading performance.

## VI. CONCLUSION

This case study reported the knee tissue resistance collected from 3 participants using a wearable system over 7 days of free-living. This wearable system utilized commercially available flexible PCBs to interface the rigid sensor system to the tissue to evaluate flex PCB performance during dynamic activity. Reliable resistance data was only reported for 1 of 3 study participants. Inspection of the flexible PCBs after use noted copper trace cracking occurring after as early as 30 hours (based on observations of degraded resistance data). This cracking was only observed at the trace-to-pad transition of the designs in both field trials and follow-up laboratory testing. While flexible PCBs are an attractive option for wearable designs based on their low-cost, quick fabrication, and accessible design (using existing CAD tools) they require further effort to develop solutions that provide reliable long term performance and methods to identify failure and degraded data to support their robust data reporting in free-living conditions.

## References

[1] Zheng Y-L , “Unobtrusive sensing and wearable devices for health informatics,” IEEE Trans. Biomed. Eng, vol. 61, no. 5, pp. 1538–1554, 5 2014. 24759283 10.1109/TBME.2014.2309951 PMC7176476

[2] Dias D and Cunha JPS, “Wearable health devices—Vital sign monitoring, systems and technologies,” Sensors, vol. 18, no. 8, p. 2414, 2018. 10.3390/s18082414 PMC6111409 30044415

[3] Patel S, Park H, Bonato P, Chan L, and Rodgers M, “A review of wearable sensors and systems with application in rehabilitation,” J. Neuroeng. Rehabil, vol. 9, no. 1, pp. 1–17, 2012. 22520559 10.1186/1743-0003-9-21 PMC3354997

[4] Düking P, Hotho A, Holmberg H-C, Fuss FK, and Sperlich B, “Comparison of non-invasive individual monitoring of the training and health of athletes with commercially available wearable technologies,” Front. Physiol, vol. 7, p. 71, 3. 2016. 27014077 10.3389/fphys.2016.00071 PMC4783417

[5] Rodríguez-Martín D, Pérez-López C, Samà A, Cabestany J, and Català A, “A wearable inertial measurement unit for long-term monitoring in the dependency care area,” Sensors, vol. 13, no. 10, pp. 14079–14104, 2013. 24145917 10.3390/s131014079 PMC3859110

[6] Kuhn H, Kimbrell W, Fowler J, and Barry C, “Properties and applications of conductive textiles,” Synth. Metals, vol. 57, no. 1, pp. 3707–3712, 1993.

[7] Jia J , “Conductive thread-based textile sensor for continuous perspiration level monitoring,” Sensors, vol. 18, no. 11, p. 3775, 2018. 10.3390/s18113775 PMC6263898 30400608

[8] Matsuhisa N, Chen X, Bao Z, and Someya T, “Materials and structural designs of stretchable conductors,” Chem. Soc. Rev, vol. 48, no. 11, pp. 2946–2966, 2019. 31073551 10.1039/c8cs00814k

[9] Kim H , “Smart patch for skin temperature: Preliminary study to evaluate psychometrics and feasibility,” Sensors, vol. 21, no. 5, p. 1855, 2021. 33800920 10.3390/s21051855 PMC7961890

[10] Du P , “High-resolution mapping of in vivo gastrointestinal slow wave activity using flexible printed circuit board electrodes: Methodology and validation,” Ann. Biomed. Eng, vol. 37, no. 4, pp. 839–846, 2009. 19224368 10.1007/s10439-009-9654-9 PMC4090363

[11] Moin A , “An EMG gesture recognition system with flexible high-density sensors and brain-inspired high-dimensional classifier,” in Proc. IEEE Int. Symp. Circuits Syst. (ISCAS), Florence, Italy, 2018, pp. 1–5.

[12] Tajima R , “Truly wearable display comprised of a flexible battery, flexible display panel, and flexible printed circuit,” J. Soc. Inf. Display, vol. 22, no. 5, pp. 237–244, 2014.

[13] Chung M, Fortunato G, and Radacsi N, “Wearable flexible sweat sensors for healthcare monitoring: A review,” J. Roy. Soc. Interface, vol. 16, no. 159, 2019, Art. no. 20190217. 10.1098/rsif.2019.0217 PMC6833321 31594525

[14] Salim A and Lim S, “Recent advances in noninvasive flexible and wearable wireless biosensors,” Biosens. Bioelectron, vol. 141, 9. 2019, Art. no. 111422. 10.1016/j.bios.2019.111422 31229794

[15] Merchant HD, Minor MG, and Liu YL, “Mechanical fatigue of thin copper foil,” J. Electron. Mater, vol. 28, no. 9, pp. 998–1007, 1999.

[16] Martynenko E, Zhou W, Chudnovsky A, Li RS, and Poglitsch L, “High cycle fatigue resistance and reliability assessment of flexible printed circuitry,” J. Electron. Packag, vol. 124, no. 3, pp. 254–259, 2002.

[17] Susan DF, Beck DF, Sorensen NR, and Thayer GE, “Fatigue behavior of thin Cu foils and Cu/Kapton flexible circuits,” Sandia Nat. Lab.(SNL-NM), Albuquerque, NM, USA, Sandia Rep. SAND2008-4293C, 2008.

[18] Wang H-F, Yang P, and Bao B-Z, “Life predict and simulation of the copper wire in flexible printed circuit board,” in Proc. 3rd Int. Conf. Meas. Technol. Mechatronics Autom, vol. 2, 2011, pp. 470–472.

[19] Naranjo-Hernández D, Reina-Tosina J, and Min M, “Fundamentals, recent advances, and future challenges in bioimpedance devices for healthcare applications,” J. Sens, vol. 2019, 7. 2019, Art. no. 9210258.

[20] Dean DA, Ramanathan T, Machado D, and Sundararajan R, “Electrical impedance spectroscopy study of biological tissues,” J. Electrostat, vol. 66, nos. 3–4, pp. 165–177, 2008. 19255614 10.1016/j.elstat.2007.11.005 PMC2597841

[21] Fu B and Freeborn TJ, “Biceps tissue bioimpedance changes from isotonic exercise-induced fatigue at different intensities,” Biomed. Phys. Eng. Exp, vol. 4, no. 2, 2018, Art. no. 025037.

[22] Freeborn TJ, Regard G, and Fu B, “Localized bicep tissue bioimpedance alterations following eccentric exercise in healthy young adults,” IEEE Access, vol. 8, pp. 23100–23109, 2020.

[23] Fu B and Freeborn TJ, “Cole-impedance parameters representing biceps tissue bioimpedance in healthy adults and their alterations following eccentric exercise,” J. Adv. Res, vol. 25, pp. 285–293, 9. 2020. 32922994 10.1016/j.jare.2020.05.016 PMC7474209

[24] Zhu F , “Estimation of normal hydration in dialysis patients using whole body and calf bioimpedance analysis,” Physiol. Meas, vol. 32, no. 7, pp. 887–902, 2011. 21646705 10.1088/0967-3334/32/7/S12

[25] Hersek S , “Wearable vector electrical bioimpedance system to assess knee joint health,” IEEE Trans. Biomed. Eng, vol. 64, no. 10, pp. 2353–2360, 10. 2017. 28026745 10.1109/TBME.2016.2641958 PMC5509509

[26] Mabrouk S, Whittingslow D, and Inan OT, “Robust method for mid-activity tracking and evaluation of ankle health post-injury,” IEEE Trans. Biomed. Eng, vol. 68, no. 4, pp. 1341–1350, 4. 2021. 32997618 10.1109/TBME.2020.3027477 PMC8034603

[27] Huynh TH, Jafari R, and Chung W-Y, “An accurate bioimpedance measurement system for blood pressure monitoring,” Sensors, vol. 18, p. 2095, 6. 2018. 10.3390/s18072095 PMC6069087 29966304

[28] Blanco-Almazán D, Groenedaal W, Catthoor F, and Jané R, “Wearable bioimpedance measurement for respiratory monitoring during inspiratory loading,” IEEE Access, vol. 7, pp. 89487–89496, 2019.

[29] Bogónez-Franco P, Nescolarde L, McAdams E, and Rosell-Ferrer J, “Multifrequency right-side, localized and segmental BIA obtained with different bioimpedance analysers,” Physiol. Meas, vol. 36, no. 1, pp. 85–106, 2014. 25501588 10.1088/0967-3334/36/1/85

[30] Mabrouk S , “Robust longitudinal ankle edema assessment using wearable bioimpedance spectroscopy,” IEEE Trans. Biomed. Eng, vol. 67, no. 4, pp. 1019–1029, 4. 2020. 31295102 10.1109/TBME.2019.2927807 PMC7217444

[31] Teague CN , “A wearable, multimodal sensing system to monitor knee joint health,” IEEE Sensors J, vol. 20, no. 18, pp. 10323–10334, 9. 2020.

[32] Márquez JC, Seoane F, and Lindecrantz K, “Textrode functional straps for bioimpedance measurements-experimental results for body composition analysis,” Eur. J. Clin. Nutr, vol. 67, no. 1, pp. S22–S27, 2013. 23299868 10.1038/ejcn.2012.161

[33] Ferreira J, Pau I, Lindecrantz K, and Seoane F, “A handheld and textile-enabled bioimpedance system for ubiquitous body composition analysis. An initial functional validation,” IEEE J. Biomed. Health Inform, vol. 21, no. 5, pp. 1224–1232, 9. 2017. 28113962 10.1109/JBHI.2016.2628766

[34] McIlduff C, Yim S, Pacheck A, Geisbush T, Mijailovic A, and Rutkove SB, “An improved electrical impedance myography (EIM) tongue array for use in clinical trials,” Clin. Neurophysiol, vol. 127, no. 1, pp. 932–935, 2016. 26242815 10.1016/j.clinph.2015.06.021 PMC4698239

[35] Seppä V-P, Väisänen J, Kauppinen P, Malmivuo J, and Hyttinen J, “Measuring respirational parameters with a wearable bioimpedance device,” in Proc. 13th Int. Conf. Elect. Bioimpedance 8th Conf. Elect. Impedance Tomogr., 2007, pp. 663–666.

[36] Moschou D, Trantidou T, Regoutz A, Carta D, Morgan H, and Prodromakis T, “Surface and electrical characterization of Ag/AgCL pseudo-reference electrodes manufactured with commercially available PCB technologies,” Sensors, vol. 15, no. 8, pp. 18102–18113, 2015. 26213940 10.3390/s150818102 PMC4570309

[37] Kusche R, Kaufmann S, and Ryschka M, “Dry electrodes for bioimpedance measurements—Design, characterization and comparison,” Biomed. Phys. Eng. Exp, vol. 5, no. 1, 2018, Art. no. 015001.

[38] Anastasova S, Kassanos P, and Yang G-Z, “Multi-parametric rigid and flexible, low-cost, disposable sensing platforms for biomedical applications,” Biosens. Bioelectron, vol. 102, pp. 668–675, 4. 2018. 29128261 10.1016/j.bios.2017.10.038

[39] Critcher S and Freeborn TJ, “Short-term evaluation of dry electrodes fabricated using flexible printed circuits processes for bioimpedance measurements,” in Proc. SoutheastCon, Raleigh, NC, USA, 2020, pp. 1–8.

[40] Bozkurt A and Lal A, “Low-cost flexible printed circuit technology based microelectrode array for extracellular stimulation of the invertebrate locomotory system,” Sens. Actuat. A, Phys, vol. 169, no. 1, pp. 89–97, 2011.

[41] Critcher S and Freeborn TJ, “Localized bioimpedance measurements with the MAX3000x integrated circuit: Characterization and demonstration,” Sensors, vol. 21, no. 9, p. 3013, 2021. 33923037 10.3390/s21093013 PMC8123364

[42] Critcher S and Freeborn TJ, “Multi-site impedance measurement system based on MAX30001 integrated-circuit,” in Proc. IEEE 63rd Int. Midwest Symp. Circuits Syst. (MWSCAS), Springfield, MA, USA, 2020, pp. 381–386.

[43] Critcher S and Freeborn TJ, “Residual impedance impact on MAX30001 accuracy for bioimpedance applications,” in Proc. 12th IEEE Latin Amer. Symp. Circuits Syst. (LASCAS), Arequipa, Peru, 2021, pp. 1–4.

[44] Kortman HGJ, Wilder SC, Geisbush TR, Narayanaswami P, and Rutkove SB, “Age- and gender-associated differences in electrical impedance values of skeletal muscle,” Physiol. Meas, vol. 34, pp. 1611–1622, 2013. 24165434 10.1088/0967-3334/34/12/1611 PMC3895401

[45] Nescolarde L, Yanguas J, Lukaski H, Alomar X, Rosell-Ferrer J, and Rodas G, “Localized bioimpedance to assess muscle injury,” Physiol. Meas, vol. 34, no. 2, pp. 237–245, 2013. 23354019 10.1088/0967-3334/34/2/237

[46] Honorato RDC, Ferraz ASM, Kassiano W, Carvalho DP, and Ceccatto VM, “Test-retest reliability of electrical impedance myography in hamstrings of healthy young men,” J. Electromyogr. Kinesiol, vol. 56, 2. 2021, Art. no. 102511. 10.1016/j.jelekin.2020.102511 33454538

[47] Lall P, Narangaparambil J, Abrol A, Leever B, and Marsh J, “Development of test protocols for the flexible substrates in wearable applications,” in Proc. 17th IEEE Intersoc. Conf. Thermal Thermomech. Phenom. Electron. Syst. (ITherm), 2018, pp. 1120–1127.

[48] Lall P, Jang H, Hill C, and Creel L, “Reliability of flexible wearable band with printed sensors for vital sign acquisition,” in Proc. Int. Techn. Conf. Exhibit. Packag. Integr. Electron. Photon. Microsyst, 10. 2020, p. 8.

[49] Fenech M and Jaffrin MY, “Extracellular and intracellular volume variations during postural change measured by segmental and wrist-ankle bioimpedance spectroscopy,” IEEE Trans. Biomed. Eng, vol. 51, no. 1, pp. 166–175, 1. 2004. 14723506 10.1109/TBME.2003.820338

